# Sweetened beverages, snacks and overweight: findings from the Young Lives cohort study in Peru

**DOI:** 10.1017/S1368980018000320

**Published:** 2018-03-20

**Authors:** Claudia Alviso-Orellana, Dayna Estrada-Tejada, Rodrigo M Carrillo-Larco, Antonio Bernabé-Ortiz

**Affiliations:** 1 School of Medicine, Universidad Peruana de Ciencias Aplicadas (UPC), Av. Alameda San Marcos, Chorrillos 15067, Lima, Peru; 2 CRONICAS Center of Excellence in Chronic Diseases, Universidad Peruana Cayetano Heredia, Lima, Peru; 3 Department of Epidemiology and Biostatistics, School of Public Health, Imperial College London, London, UK; 4 Faculty of Epidemiology and Population Health, London School of Hygiene and Tropical Medicine, London, UK

**Keywords:** Snacks, Sweetened beverages, Overweight, Children

## Abstract

**Objective:**

To determine the association between consumption of snacks and sweetened beverages and risk of overweight among children.

**Design:**

Secondary analysis of the Young Lives cohort study in Peru.

**Setting:**

Twenty sentinel sites from a total of 1818 districts available in Peru.

**Subjects:**

Children in the younger cohort of the Young Lives study in Peru, specifically those included in the third (2009) and the fourth (2013) rounds.

**Results:**

A total of 1813 children were evaluated at baseline; 49·2 % girls and mean age 8·0 (sd 0·3) years. At baseline, 3·3 (95 % CI 2·5, 4·2) % reported daily sweetened beverage consumption, while this proportion was 3·9 (95 % CI 3·1, 4·9) % for snacks. Baseline prevalence of overweight was 22·0 (95 % CI 20·1, 23·9) %. Only 1414 children were followed for 4·0 (sd 0·1) years, with an overweight incidence of 3·6 (95 % CI 3·1, 4·1) per 100 person-years. In multivariable analysis, children who consumed sweetened beverages and snacks daily had an average weight increase of 2·29 (95 % CI 0·62, 3·96) and 2·04 (95 % CI 0·48, 3·60) kg more, respectively, than those who never consumed these products, in approximately 4 years of follow-up. Moreover, there was evidence of an association between daily consumption of sweetened beverages and risk of overweight (relative risk=2·12; 95 % CI 1·05, 4·28).

**Conclusions:**

Daily consumption of sweetened beverages and snacks was associated with increased weight gain *v*. never consuming these products; and in the case of sweetened beverages, with higher risk of developing overweight.

Excess weight is currently one of the main health concerns worldwide: the prevalence of obesity has increased from 3·2 % among adult men and 6·4 % among adult women in 1975, to 10·8 and 14·9 % in 2014, respectively^(^ ^1^ ^)^. Moreover, between 1975 and 2016, the prevalence of obesity has increased by fivefold and sevenfold, respectively, in girls and boys aged 5–19 years^(^ ^2^ ^)^. On the other hand, the prevalence of overweight ranges from 18·9 to 36·9 % in school-aged children (5–11 years) and from 16·6 to 35·8 % in adolescents (12–19 years) in Latin America^(^ ^3^ ^)^. In Peru, the prevalence of overweight was 29·4 % among children aged 5–9 years according to a nationally representative sample. This figure was 24·2 % among adolescents^(^ ^4^ ^)^.

Research has demonstrated that children with overweight have a higher probability of continuing with that condition throughout adolescence and adulthood^(^ ^5^ ^)^, increasing the risk of developing metabolic syndrome, type 2 diabetes mellitus, CVD and mortality^(^ ^6^ ^)^. Moreover, several factors have been involved in the development of overweight, including genetic factors^(^ ^7^ ^)^, sociocultural influences^(^ ^8^ ^)^, as well as socio-economic background^(^ ^9^ ^)^. Diet patterns, mainly the consumption of high-energy-dense foods (i.e. unhealthy snacks, junk foods and sweetened beverages), are also involved in the development of overweight^(^ ^10^ ^–^ ^16^ ^)^. Snacks and sweetened beverages are an important target in the prevention of overweight as they are modifiable components of diet^(^ ^17^ ^)^. Nevertheless, the magnitude at which the consumption of snacks and sweetened beverages leads to obesity has to be better characterized in countries where their consumption is on the rise.

According to the FAO, snacks are ‘foods that are eaten between main meals and have a tendency to have a lower nutritional value’^(^ ^18^ ^)^. Snacks are available in different presentations such as fruits, vegetables and nuts, but there are also snacks with high energy content (i.e. soft drinks, sweets and junk foods). Thus, the present study aimed to determine the association between the consumption of unhealthy foods, mainly snacks and sweetened beverages (i.e. soft drinks), and the prevalence and incidence of overweight in Peru, a middle-income country undergoing the nutrition transition. For this, data from the Young Lives cohort study were analysed.

## Methods

### Study design

The present study is a secondary analysis using data of the younger cohort of children enrolled in the Young Lives study^(^ ^19^ ^)^ which is conducted in four developing countries: Ethiopia, India, Peru and Vietnam. The Young Lives study started in 2002 and continues to date; it comprises questionnaires on nutrition and health and includes anthropometric measurements. Details of the study have been published previously^(^ ^19^ ^)^ and the data are freely available online (http://www.younglives.org.uk).

### Study population

The Young Lives study includes two longitudinal prospective cohorts, a younger and an older one. At baseline, in the year 2002, the younger cohort included children aged 6 to 18 months old, whereas the older cohort included children aged between 7 and 8 years old. For the present paper, we utilized data from the third (2009) and the fourth round (2013) of the younger cohort in Peru. Data of children aged 7–8 years in the third round were considered our baseline (attrition rate was 1·1 %^(^ ^19^ ^)^), whereas information from the fourth round was also included for the follow-up outcomes. Participants with incomplete data in the variables of interest (i.e. overweight/obesity, snacks and sweetened beverages) were excluded from the analysis.

### Sampling and procedures

The sampling approach for the Peruvian cohort has been broadly explained elsewhere^(^ ^20^ ^)^. Briefly, a sentinel site sampling approach was followed using a multistage, cluster-stratified, random sampling technique. The initial sample frame at the district level was used to choose twenty sentinel sites from a total of 1818 districts available. The top 5 % richest districts were excluded because the aim was to oversample poor areas. Once the districts were selected, maps of census tracts were used to randomly choose one census tract in each district using a random number table. Blocks of houses and sets of houses were selected per district. Finally, all households in any given block or set of houses were visited to identify one household with at least one child for the study purposes. Blocks of houses or sets of houses were approached until the total eligible households were found. For data collection, three teams, comprising fieldworkers, a data-entry clerk and supervisors, were responsible for six or seven sentinel sites.

### Study variables

#### Outcome

The outcome of interest was child overweight, based on BMI. The International Obesity Task Force’s sex- and age-specific BMI cut-off points for children were used for analysis^(^ ^21^ ^)^. We decided to use the International Obesity Task Force criteria because they give more conservative estimates of overweight compared with other international definitions^(^ ^22^ ^)^. In addition, weight (in kilograms) and circumference abdominal (in centimetres) were also outcomes of interest but considered as numerical variables.

#### Exposures

Exposures were consumption of snacks and consumption of sweetened beverages, which were assessed using the following questions: ‘During the last 30 d, did the child (name) eat salty and fatty foods, such as crisps or fried snacks?’ and ‘During the last 30 d, did the child (name) drink fizzy, sweet soft drinks such as sodas?’ Responses were given by the child’s mother with six options, including: ‘never’, ‘less than every 2 weeks’, ‘every 2 weeks’, ‘once a week’, ‘2–6 times a week’ and ‘daily’. For analysis purposes, the responses were categorized as never (reference), up to every 2 weeks, 2–6 times per week and daily.

#### Covariates

Other variables, assessed at baseline, also included in the analyses as potential confounders were: age (7 *v*. 8 years), sex (male *v*. female), socio-economic status (based on a wealth index built using household assets and facilities, and split in tertiles) and physical activity (assessed by report of the child’s mother of at least 60 min of physical activity on none, 1–3, 4–6 and 7 d per week). In addition, mother’s overweight status (<25 *v*. ≥25 kg/m^2^) was also included.

### Statistical analysis

The statistical software package STATA version 13 for Windows was used for analysis. Initially, characteristics of the study population according to snack and sweetened beverage consumption were tabulated. Then, change in weight, BMI and waist circumference were determined by the difference between estimates at follow-up (round 4) and baseline (round 3) using linear regression models. In addition, multivariable linear regression models were built to assess the association of interest with numerical outcomes.

Further, the incidence of overweight as per BMI, using the child age- and sex-specific cut-off points proposed by the International Obesity Task Force^(^ ^21^ ^)^, was estimated after excluding cases of overweight at baseline and reported per 100 person-years of follow-up. Finally, Poisson regression models, with link log and robust standard errors, were used to estimate the strength of the association between exposure and outcomes, reporting relative risks (RR) and 95 % CI.

### Ethics

The Young Lives study was originally approved by the Ethics Committee Social Science Division, University of Oxford, UK, in the year 2006. In Peru, the approval was granted by the Research Ethics Committee at the Instituto de Investigacion Nutricional in Lima. To conduct the current secondary analysis, ethical approval was obtained from the Ethical Committee at the Universidad Peruana de Ciencias Aplicadas (UPC), in Lima, Peru.

## Results

### Population characteristics at baseline

**Fig. 1:**
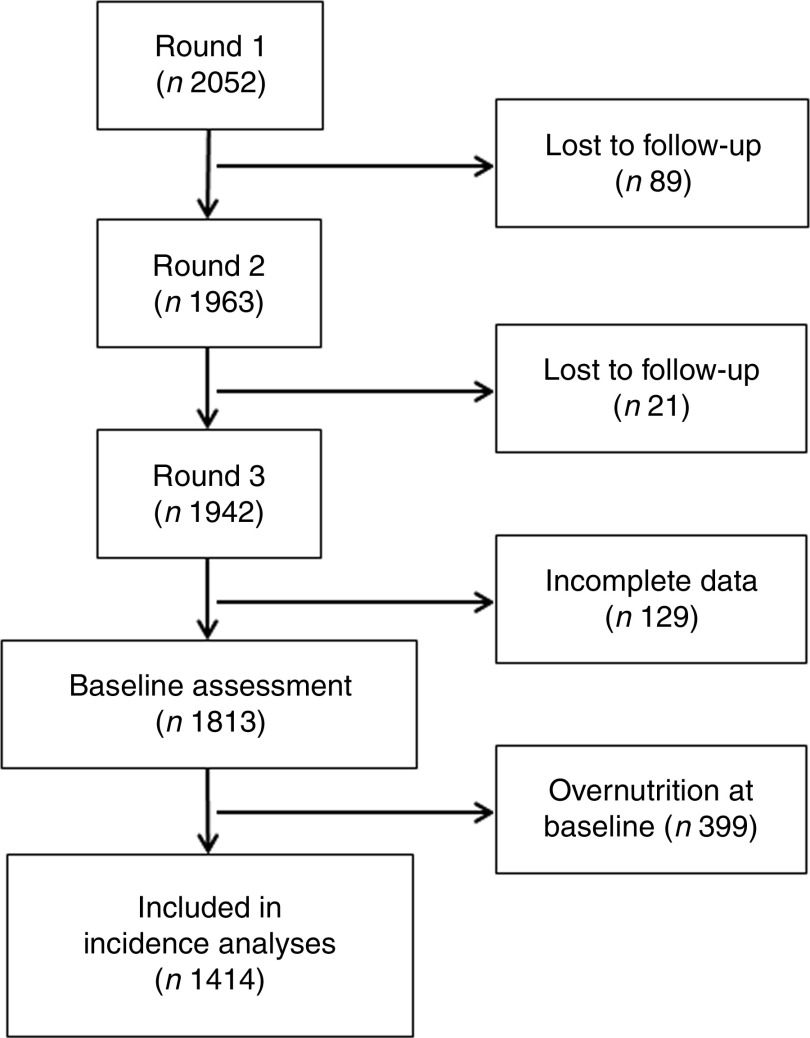
Flowchart of participants in the Young Lives study. Round 3 of the Young Lives study was considered baseline in the current analysis

In total 1942 children were assessed in the third round (Fig. 1); of them, 129 were excluded because of incomplete information on the variables of interest, and as a result data from 1813 children were analysed, mean age 8·0 (sd 0·3) years and 49·2 % were girls.

A total of 12·9 (95 % CI 11·3, 14·5) % reported never having consumed sweetened beverages, whereas 3·3 (95 % CI 2·5, 4·2) % reported consuming them daily. Similarly, corresponding values were 20·0 (95 % CI 18·2, 21·9) % and 3·9 (95 % CI 3·1, 4·9) % for snack consumption. Overweight was present in 22·0 (95 % CI 20·1, 23·9) % of children. Information on the study population characteristics by sweetened beverage and snack consumption is detailed in Table 1.

**Table 1:** Baseline description of the study population by exposure (sweetened beverage and snack consumption): children from the younger cohort of the Young Lives cohort study in Peru, who participated in the third (2009; considered baseline in the current analysis) and the fourth (2013; follow-up) rounds

			Sweetened beverage consumption				Snack consumption			
	Total (*n* 1813)		Never (*n* 233)	Up to every 2 weeks (*n* 577)	2–6 times per week (*n* 944)	Daily (*n* 59)	Never (*n* 363)	Up to every 2 weeks (*n* 577)	2–6 times per week (*n* 944)	Daily (*n* 71)
Variable	*n*	%	%	%	%	%	%	%	%	%
Sex										
Female	892	49·2	48·5	48·4	49·5	55·9	50·1	46·8	50·5	46·5
Male	921	50·8	51·5	51·6	50·5	44·1	49·9	53·2	49·5	53·5
Age										
7 years	973	53·7	54·9	50·4	55·1	55·9	55·1	53·4	54·3	40·9
8 years	840	46·3	45·1	49·6	44·8	44·1	44·9	46·6	45·7	59·2
Wealth index										
Low	601	33·3	57·1	40·7	23·3	22·0	55·9	32·7	24·9	18·3
Middle	598	33·4	28·8	30·5	35·7	30·5	28·9	31·6	35·1	39·4
High	614	33·3	14·2	28·8	41·0	47·5	15·2	35·7	40·0	42·3
Physical activity										
None/week	169	9·3	14·6	6·6	9·7	10·2	13·2	7·5	9·2	4·2
1–3 d/week	865	47·7	41·6	52·3	46·6	45·8	43·8	54·0	45·9	43·7
4–6 d/week	181	10·0	11·6	10·6	9·4	6·8	9·9	10·5	10·3	2·8
7 d/week	597	33·0	32·2	30·5	34·4	37·3	33·1	28·0	34·6	49·3
Mother’s BMI										
Overweight	1108	66·1	53·1	63·6	70·0	72·2	59·2	69·4	66·5	71·6
Child BMI										
Overweight	399	22·0	14·6	20·5	24·5	27·1	11·6	22·6	26·6	16·9
Child waist circumference										
Mean	60·7		59·3	60·7	60·9	61·5	58·9	60·7	61·4	61·8
sd	6·2		5·0	6·3	6·3	6·8	4·8	6·2	6·5	6·4

Results may not add due to missing values.

### Change in BMI, weight and waist circumference over time

Overall, the mean weight of the population increased by 15·9 (sd 6·1) kg during the 4 years of follow-up. Similarly, the mean BMI increased by 2·7 (sd 2·2) kg/m^2^, whereas the mean waist circumference rose by 8·6 (sd 6·5) cm. In multivariable models, children reporting daily consumption of sweetened beverages had a higher weight increase than those who never consumed them (2·29 kg; 95 % CI 0·62, 3·96 kg). Similarly, there was a higher weight gain (2·04 kg; 95 % CI 0·48, 3·60 kg) among those consuming snacks daily (Table 2). The same trend was also observable when BMI was the outcome. However, changes in waist circumference were not significant for sweetened beverages (1·43 cm; 95 % CI −0·41, 3·27 cm) or snacks (0·85 cm; 95 % CI −0·89, 2·60 cm).

**Table 2:** Change in weight, BMI and waist circumference during follow-up by exposure (sweetened beverage and snack consumption) among children from the younger cohort of the Young Lives cohort study in Peru, who participated in the third (2009; considered baseline in the current analysis) and the fourth (2013; follow-up) rounds

			Crude model		Adjusted model†	
Outcome	Exposure	Category*	Coefficient	95 % CI	Coefficient	95 % CI
Weight (kg)	Sweetened beverages	Up to every 2 weeks	**1·61**	**0·77, 2·46**	0·58	−0·22, 1·37
		2–6 times per week	**2·44**	**1·65, 3·24**	0·55	−0·21, 1·32
		Daily	**3·93**	**2·19, 5·67**	**2·29**	**0·62, 3·96**
	Snacks	Up to every 2 weeks	**1·66**	**0·89, 2·43**	0·46	−0·29, 1·20
		2–6 times per week	**2·50**	**1·79, 3·22**	**0·97**	**0·26, 1·67**
		Daily	**3·82**	**2·14, 5·50**	**2·04**	**0·48, 3·60**
BMI (kg/m^2^)	Sweetened beverages	Up to every 2 weeks	0·20	−0·10, 0·50	−0·08	−0·39, 0·24
		2–6 times per week	**0·59**	**0·31, 0·87**	0·14	−0·15, 0·43
		Daily	**1·12**	**0·53, 1·70**	**0·74**	**0·15, 1·33**
	Snacks	Up to every 2 weeks	0·20	−0·07, 0·47	−0·10	−0·38, 0·18
		2–6 times per week	**0·57**	**0·31, 0·83**	0·20	−0·08, 0·47
		Daily	**1·11**	**0·52, 1·69**	**0·71**	**0·14, 1·28**
Waist circumference (cm)	Sweetened beverages	Up to every 2 weeks	0·68	−0·11, 1·47	0·16	−0·64, 0·96
		2–6 times per week	**1·64**	**0·87, 2·40**	0·80	−0·01, 1·61
		Daily	**2·40**	**0·64, 4·17**	1·43	−0·41, 3·27
	Snacks	Up to every 2 weeks	0·66	−0·12, 1·43	−0·15	−0·95, 0·65
		2–6 times per week	**1·13**	**0·38, 1·88**	0·26	−0·52, 1·04
		Daily	1·58	−0·23, 3·38	0·85	−0·89, 2·60

Significant (*P*<0·05) estimates are presented in bold.

* All categories were compared with those who ‘never’ consumed sweetened beverages/snacks.

† Model adjusted for sex, age, socio-economic status, physical activity and mother’s BMI at baseline.

### Overweight risk with sweetened beverage and snack consumption

For the incidence analysis, 399 children were excluded as they were overweight at baseline. Thus, 1414 children were included for these estimations. Mean time of follow-up was 4·0 (sd 0·1) years, completing 5523·1 person-years of follow-up. A total of 197 new cases of overweight were found with an overall incidence of 3·57 (95 % CI 3·10, 4·10) per 100 person-years.

Although there was a clear trend of rising incidence of overweight associated with increased consumption of sweetened beverages (*P*<0·001) and snacks (*P*=0·003), in multivariable models, only daily sweetened beverage consumption was associated with higher risk of becoming overweight (RR=2·12; 95 % CI 1·05, 4·28). Details of the regression models are shown in Table 3.

**Table 3:** Association of sweetened beverage and snack consumption with overweight (incidence, crude and adjusted models) among children from the younger cohort of the Young Lives cohort study in Peru, who participated in the third (2009; considered baseline in the current analysis) and the fourth (2013; follow-up) rounds

		Incidence per 100 person-years		Crude model		Adjusted model*	
Exposure	Category	RR	95 % CI	RR	95 % CI	RR	95 % CI
Sweetened beverage consumption	Never	1·94	1·17, 3·21	1·00	Reference	1·00	Reference
	Up to every 2 weeks	3·01	2·30, 3·93	1·55	0·90, 2·68	1·15	0·66, 1·99
	2–6 times per week	4·20	3·51, 5·04	**2·16**	**1·29, 3·61**	1·34	0·80, 2·25
	Daily	6·54	3·62, 11·82	**3·46**	**1·72, 6·97**	**2·12**	**1·05, 4·28**
Snack consumption	Never	2·41	1·68, 3·44	1·00	Reference	1·00	Reference
	Up to every 2 weeks	3·09	2·34, 4·07	1·29	0·84, 1·98	0·99	0·64, 1·54
	2–6 times per week	4·36	3·61, 5·28	**1·81**	**1·23, 2·64**	1·29	0·87, 1·92
	Daily	4·83	2·68, 8·73	**2·00**	**1·06, 3·75**	1·43	0·76, 2·69

RR, relative risk. Significant (*P*<0·05) estimates are presented in bold.

* Model adjusted for sex, age, socio-economic status, physical activity and mother’s BMI at baseline.

## Discussion

### Main findings

Our results revealed that in resource-limited settings in Peru, four in 100 children per year developed overweight. Moreover, daily consumption of sweetened beverages, compared with never consuming them, doubled the risk of developing overweight. These results emphasize the relevance of working to restrict consumption of sweetened beverages, particularly for children.

### Comparison with previous studies

Previous studies have reported the association between sweetened beverage as well as snack consumption and developing overweight among children^(^ ^11^ ^–^ ^16^ ^)^. Previous prospective studies have reported that soft drinks and snacks can increase fat mass in children^(^ ^23^ ^)^, the risk of metabolic syndrome and high blood pressure levels^(^ ^13^ ^,^ ^24^ ^)^, and CVD^(^ ^10^ ^)^. Our results confirm the deleterious effect of unhealthy foods, mainly sweetened beverages, on children’s health; but also add evidence from resource-constrained settings undergoing the nutrition transition.

Possible mechanisms to explain these associations have been pointed out by previous clinical studies. A clinical trial assessing the impact of sweetened beverages on adults’ health^(^ ^25^ ^)^ found that the daily intake of sweetened drinks increased ectopic fat and lipid accumulation compared with milk or water, enhancing the risk of cardiovascular and metabolic diseases. This process was mainly associated with fructose, which can enhance *de novo* lipogenesis, TAG production and fat accumulation in the liver^(^ ^26^ ^)^.

It is most likely that parents decide what the young household members eat, meaning that children may not independently decide what to eat^(^ ^27^ ^,^ ^28^ ^)^. This is important because our findings show that 65 % of mothers were overweight. A recent systematic review found that both healthy and unhealthy parental behaviours are strongly correlated with child food consumption behaviour^(^ ^29^ ^)^. Then, addressing parents’ behaviours could indirectly positively impact their children as well.

### Public health relevance

The rise of overweight among children has become an important public health problem^(^ ^2^ ^)^. Moreover, of the 184 000 deaths per year due to sweetened beverage consumption worldwide, 75·9 % occur in low- and middle-income countries^(^ ^17^ ^)^, and Latin America and the Caribbean have the highest absolute mortality related to sweetened beverages.

Snack and sweetened beverage consumption are modifiable components of diet. Although overweight prevalence among children in Peru is low compared with other Latin American countries, our results indicate incidence rates comparable to those observed in adults^(^ ^30^ ^)^. This suggests that if risk factors, including consumption of sweetened beverages, are not reduced, then the prevalence of childhood obesity may reach that of other countries in the region. Moreover, it has been described that the typical Peruvian diet is high in carbohydrates and low in fruits and vegetables^(^ ^31^ ^)^. As it could be harder to change inherited characteristics of the Peruvian diet, we would strongly suggest dedicating efforts to tackle relatively new risk factors such as high snack and soda consumption.

The high correlation between parental behaviour and child food consumption may be used to implement appropriate interventions. For example, a study conducted in Peru demonstrated that an integral programme promoting healthy behaviours among parents (lectures on energy balance and healthy snacking), nutritional counselling to food providers in school and promoting physical activity contributed to the reduction of BMI in children^(^ ^32^ ^)^.

From the policy and legal perspective, taxation can be a good strategy to tackle overweight. An increment of taxes on soda has shown a significant reduction of obesity, BMI and type 2 diabetes mellitus, especially in areas with greater prevalence of obesity and in resource-constrained settings^(^ ^33^ ^–^ ^37^ ^)^. Thus, our study supports the need for addressing the problem of overweight, especially among children and adolescents, from different perspectives.

### Strengths and limitations

Strengths of the present study include the use of data from a prospective ongoing study, reducing the risk of reverse causality. In addition, a low proportion of individuals were lost to follow-up; thus, the impact of the attrition rate can be considered negligible^(^ ^19^ ^)^. However, the study has several limitations. First, our exposures were based on self-report data reported by a third person (i.e. child’s mother) and as a result, the exact frequency of sweetened beverage and snack intake could not be obtained. In addition, recall and desirability bias can be an issue when trying to remember food consumption. If so, the consumption could have been even higher, making our results conservative of a possible stronger effect. Second, the question used to assess sweetened beverage consumption was not specific to sodas, but potentially also included diet sodas, sports drinks, etc. However, artificially sweetened beverages have also been linked to increased adiposity, type 2 diabetes mellitus and other detrimental health effects^(^ ^38^ ^,^ ^39^ ^)^. Third, we assumed that information about snack and sweetened beverage consumption in the 30 d prior to the survey is indicative of long-term consumption. Finally, other variables, potential confounders in our association of interest, were not available for the analysis, such as parents’ educational level, fruit and vegetable intake, among others.

## Conclusions

The daily consumption of sweetened beverages and snacks was associated with increased weight and overweight among children. Our results suggest that interventions to reduce the consumption of these products are needed to tackle the obesity epidemic in Peru and other similar countries.
